# Targeting Androgen, Thyroid Hormone, and Vitamin A and D Receptors to Treat Prostate Cancer

**DOI:** 10.3390/ijms25179245

**Published:** 2024-08-26

**Authors:** Brigitte Hantusch, Lukas Kenner, Vesna S. Stanulović, Maarten Hoogenkamp, Geoffrey Brown

**Affiliations:** 1 Department of Pathology, Department for Experimental and Laboratory Animal Pathology, Medical University of Vienna, 1010 Vienna, Austria; brigitte.hantusch@meduniwien.ac.at; 2 Comprehensive Cancer Center, Medical University Vienna, 1090 Vienna, Austria; 3 Unit of Laboratory Animal Pathology, University of Veterinary Medicine Vienna, 1210 Vienna, Austria; 4 Department of Molecular Biology, Umeå University, 901 87 Umeå, Sweden; 5 Christian Doppler Laboratory for Applied Metabolomics, Medical University Vienna, 1090 Vienna, Austria; 6 Center for Biomarker Research in Medicine (CBmed), 8010 Graz, Austria; 7 Institute of Cancer and Genomic Sciences, College of Medical and Dental Sciences, University of Birmingham, Edgbaston, Birmingham B15 2TT, UK; v.stanulovic@bham.ac.uk (V.S.S.); m.hoogenkamp@bham.ac.uk (M.H.); 8 School of Biomedical Sciences, Institute of Clinical Sciences, College of Medical and Dental Sciences, University of Birmingham, Edgbaston, Birmingham B15 2TT, UK

**Keywords:** prostate cancer, nuclear hormone receptors, androgen receptor, thyroid hormone receptor, retinoic acid receptor, vitamin D receptor

## Abstract

The nuclear hormone family of receptors regulates gene expression. The androgen receptor (AR), upon ligand binding and homodimerization, shuttles from the cytosol into the nucleus to activate gene expression. Thyroid hormone receptors (TRs), retinoic acid receptors (RARs), and the vitamin D receptor (VDR) are present in the nucleus bound to chromatin as a heterodimer with the retinoid X receptors (RXRs) and repress gene expression. Ligand binding leads to transcription activation. The hormonal ligands for these receptors play crucial roles to ensure the proper conduct of very many tissues and exert effects on prostate cancer (PCa) cells. Androgens support PCa proliferation and androgen deprivation alone or with chemotherapy is the standard therapy for PCa. RARγ activation and 3,5,3′-triiodo-L-thyronine (T3) stimulation of TRβ support the growth of PCa cells. Ligand stimulation of VDR drives growth arrest, differentiation, and apoptosis of PCa cells. Often these receptors are explored as separate avenues to find treatments for PCa and other cancers. However, there is accumulating evidence to support receptor interactions and crosstalk of regulatory events whereby a better understanding might lead to new combinatorial treatments.

## 1. Introduction

Prostate cancer (PCa) is the second most common tumor in males. Hormone sensitivity is a trait Achilles heel of PCa whereby androgen deprivation therapy (ADT), which began in 1941, represents the core strategy of PCa treatment [1]. This treatment alone or with chemotherapy remains the standard therapy, but many patients progress to ADT-resistant tumors termed castration-resistant PCa (CRPC) which is mainly associated with amplifications, mutations, and gene rearrangements to the androgen receptor (AR) [2,3]. The potentiation of AR in the CRPC setting historically constituted the rationale for combining ADT with drugs that either target androgen synthesis (abiraterone) or block the AR (enzalutamide and bicalutamide), leading to a beneficial clinical outcome [4,5,6]. Other steroidal hormone receptors, including the progesterone receptor (PR) [7], estrogen receptors (ERs) [8], and the glucocorticoid receptor (GR) [9], have been implicated in PCa tumorigenesis and resistance development. 

For PCa, we examine the influence of androgens, 3,5,3′-triiodo-L-thyronine (T3) stimulation of the thyroid hormone receptor (TR) β, the use of synthetic retinoids to selectively antagonize the retinoic acid receptor (RAR) γ, and activation of the vitamin D receptor (VDR) by 1α,25-dihydroxyvitamin D3 (1,25D3), the active metabolite of vitamin D3. We focus on the activation status of these receptors because they influence the proliferation of PCa cells whereby activity of the AR, TRβ, and RARγ enhance PCa proliferation and active VDR drives growth arrest followed by apoptosis. We describe the current state of knowledge regarding the modes of action of the hormones and their receptors, particularly their interactions and the extent to which there is also regulatory crosstalk. A better appreciation of crosstalk is likely to offer new therapeutic possibilities, especially for hormone-dependent forms of cancer [10]. Our aim is to highlight the potential of targeting the above-mentioned nuclear receptors (NRs) to treat PCa. There are extensive reviews of the roles of other NRs in PCa [11] including orphan nuclear receptors (ONRs) [12].

## 2. Nuclear Hormone Receptors

### 2.1. The Functionalities

Hormones exert continuous control on gene regulation processes to ensure the proper functioning of all tissues and, therefore, the well-being of an organism. A hormonal imbalance due to an excess or a deficiency can lead to severe and sometimes life-threatening diseases. From studies of estrogen, it became clear that hormones act through NRs to regulate gene expression [13,14]. In 1985, the first full-length NR to be cloned was the human GR [15], and around the same time, the ERα was cloned [16]. Soon, it became clear that these receptors are structurally similar and belong to a NR superfamily [10,17].

In addition to NR family members binding classical hormones, they also bind lipid-soluble ligands, including the most active metabolite of vitamin A all-*trans* retinoic acid (ATRA), 1,25D3, and oxysterols. Binding activates the essential role of NRs as transcription factors (TFs). Many NRs and their isoforms have been cloned and many of their regulatory cofactors have been identified. Presently, there are 48 genes for NRs and closely related orphan NRs (ONRs), and their phylogenetic classification is based on protein sequence homologies [12,14]. They all contain a highly conserved modular structure, and the evolutionarily oldest family members function without ligands. Whilst much is known about NRs and their modes of action, information about their tissue- and disease-specific effects and their binding cistromes still needs to be discovered. Other poorly understood areas include the extent to which NRs interact and their potential mutual regulation.

### 2.2. Phylogenetical Features

**Figure 1:**
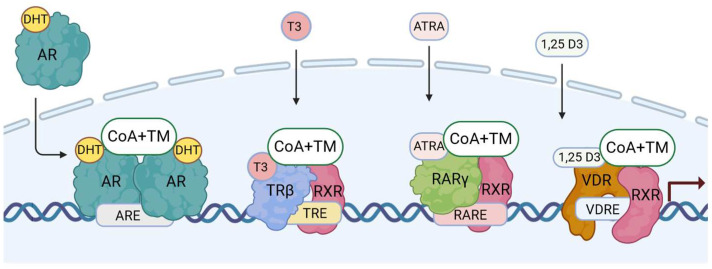
Principles of type I and type II NR-driven gene regulation. AR, androgen receptor; DHT, dihydrotestosterone; TR, thyroid receptor; T3, 3,5,3′-triiodo-L-thyronine; RAR, retinoic acid receptor; ATRA, all-*trans* retinoic acid; VDR, vitamin D receptor; 1,25D3, 1α,25-dihydroxyvitamin D3; RXR, retinoid X receptor; CoA, coactivator; TM, transcription machinery, ARE, androgen response element; TRE, thyroid response element; RARE, retinoic acid response element; VDRE, vitamin D response element. Created with BioRender.com.

NRs contain four main structural elements. They are the N-terminal domain (NTD), a gene transactivation domain which includes the DNA-binding domain (DBD), a flexible hinge region (H) which contains the nuclear localization motif (NLS), and a ligand-binding domain (LBD) [14,18,19]. NRs function as TFs in an allosteric manner, typically switching between an inactive (ligand-free) and an active (ligand-bound) form. An important feature of NRs is that they can act as monomers, homodimers, and heterodimers with other NRs [18,20]. Figure 1 shows how gene expression is driven by type I and type II NRs via canonical dimers.

**Figure 2:**
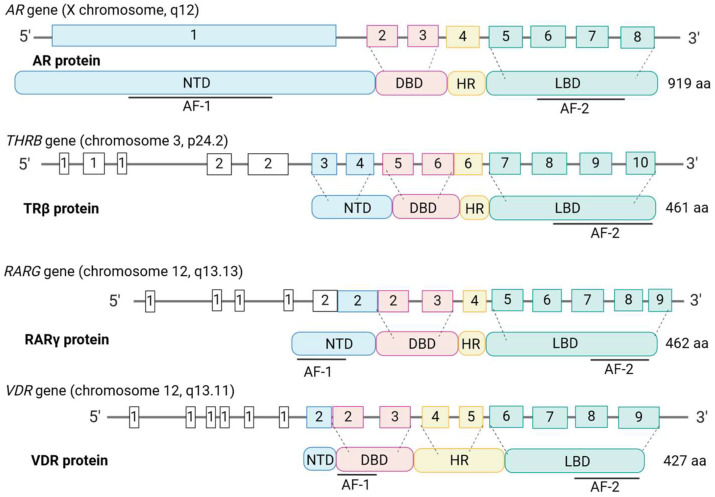
Structure of the androgen (AR), thyroid (TR), retinoic acid (RAR), and vitamin D receptor (VDR) genes and proteins. NTD, N-terminal region; DBD, DNA-binding domain; HR, hinge region; LBD, ligand binding domain; AF, activation domain. Created with BioRender.com.

The NRs are broadly divided into four types based on their functional characteristics and modes of DNA binding [20]. Type 1 NRs are in the cytosol and shuttle upon ligand-binding and homodimerization into the nucleus. They are steroid hormone-binding receptors and include the AR, the GR, the PR, the mineralocorticoid receptor (MR), and the ERs. Type II NRs include the TRs, RARs, VDR, and peroxisome proliferator-activated receptors (PPARs). They are present in the nucleus and are localized to chromatin as RXR heterodimers in a repressive mode. They become transcriptionally active when ligands are bound, which leads to the loss of corepressors and the recruitment of coactivators. Type III NRs are the homodimeric orphan receptors; they shuttle as dimers and bind to direct hormone response element (HRE) repeats. Type IV NRs are the monomeric orphan receptors [18]. The four types of NRs have been considered as a putative target regarding therapies for PCa [21]. Proteomic studies have revealed the capacity of NRs to liaise with one another and various cofactors [22], creating a wide range of NR interactome possibilities. Crystal structure analyses have revealed non-canonical dimerization and even multimeric forms [19], highlighting a high functional versatility. Figure 2 shows the structures of the AR, TR, RAR, and VDR genes and proteins.

### 2.3. DNA-Binding Motifs and Gene Expression Regulation

The NR regions relevant for DNA binding and heterodimerization were identified by inserting specific mutations, leading to the elucidation of the nucleotide nature of the DNA-binding motifs and characterization of HREs [23]. Like other TFs, how NRs interact with chromatin depends on simple, highly conserved hexamer DNA motifs. Ingeniously, their orientation and spacer regions lead to the fine tuning of binding capabilities [17,22,24]. Importantly, regarding DNA response element binding, TR, VDR, and RAR bind as heterodimers with retinoid X receptors (RXRs), including pairing with RXRα, β or γ. According to the 3-4-5 rule, these are always direct AGGTCA hexamer repeats, spaced by three nucleotides for the VDR, four nucleotides for TRs, and five nucleotides for RARs. There are other binding possibilities, including additions to the known “canonical” motifs. One site sequence would not be sufficient because a single transcription factor can recognize hundreds of DNA sequences and bind within a range of affinities, and the local DNA structure is also essential [25]. All these parameters greatly expand the potential scope of gene expression regulation [26]. To add to this complexity, there are isoform variants of NRs and their multitude interactions and ligand- and DNA-binding capabilities result in regulatory possibilities that are still barely defined [27]. Finally, NRs regulate distinct genes in different tissues, and most probably, they are different in diseases due to pathophysiological changes.

## 3. Androgen Receptor

Androgen deprivation therapy is the gold-standard treatment for most PCa patients. However, CRPC, which is a highly aggressive metastatic hormone-independent form of the disease, develops in a significant number of patients. Treatment options for these patients are limited, even with the recent introduction of improved therapeutics.

### 3.1. The Functionalities of the AR

The AR gene is located at q11-12 on the X chromosome and encodes a 920 amino acid protein. Alternative splicing results in multiple transcript variants encoding different isoforms. As for all NRs, there are four main structural elements to the protein (Figure 2). Together with the GR, PR, and MR receptors, the AR forms the oxosteroid superfamily, which is phylogenetically separated from the ERs and TRs [28]. The AR is a classical steroid hormone receptor whereby functionality is critically dependent on the presence of androgens. They are a group of steroidal sex hormones that are produced in the testes, the ovaries, and the adrenal glands and androgens are the main drivers of male sex development. Testosterone, the major androgen, is converted intracellularly to dihydrotestosterone (DHT), which has the highest affinity for the AR. Post ligand binding, the AR forms homodimers which then translocate to the nucleus [29] to control gene expression by binding to inverted repeat androgen response elements (AREs) [24].

### 3.2. AR and Prostate Cancer

The role of AR in PCa is described exhaustively in numerous reviews [30,31]. The AR maintains a healthy prostate epithelium, including its proper growth and differentiation. Overactivation alone does not lead to PCa [11], and instead, the AR plays a role in conjunction with other malignant processes. In this case, correlating AR expression levels with the risk of PCa development is not significant. 

AR contributes to PCa growth by driving the expression of genes that control cell proliferation. Whilst AR-mediated changes to PCa-associated gene expression are known, their contributions to PCa and its dissemination still need to be fully understood. There are changes in extranuclear steroid hormone receptor signaling [32], and the AR, after shuttling to the nucleus, shows altered DNA binding capabilities, which reshapes the AR-driven cistrome. Since the canonical AR binding motif and its structure are highly conserved, this must be due to chromatin landscape changes and its accessibility, as was shown for the cadherin 1 gene [33].

Whilst AR contributes to PCa growth, it is important to bear in mind that the influence of androgens is complex. LNCaP cells are widely studied as a model for androgen-dependent PCa as they express ARs at a considerable level. The dose responsiveness of LNCaP cells to DHT is biphasic. Placing LNCaP cells in charcoal-stripped serum inhibited their proliferation, and they remained viable for up to 30 days. This inhibition of growth was reversed by adding DHT at a very low concentration (3 × 10^−10^ M), but the cell yield was progressively reduced by higher concentrations. Only androgens were able to trigger this inhibition of proliferation [34]. Other workers have also reported that the androgen responsiveness of LNCaP cells is biphasic whereby the synthetic androgen R1881 stimulated growth at a low concentration (<1 nM) and inhibited growth at higher concentrations [35]. These findings led to the use of high-dose testosterone, namely bipolar androgen therapy (BAT), to treat advanced PCa that is resistant to hormone-blocking therapy [36].

A further consideration of the influence of DHT on PCa is that AR regulates the transcription of DNA repair genes. From transcriptome analyses of LNCaP cells treated with the antiandrogen ARN-509, DNA repair gene sets were enriched in the control versus treated cells. An AR-associated DNA repair signature was seen for LNCaP cells treated with the synthetic androgen R1881 (RNA-seq and Chip-seq), and antiandrogen treatment downregulated DNA repair genes. LNCaP cells that were treated with ionizing radiation plus the androgen showed decreased DNA damage and increased repair, and the antiandrogen caused opposite effects and resulted in decreased classical nonhomogeneous end-joining (C-NHEJ). These findings provide a potential mechanism to explain why androgen-deprivation therapy synergizes with ionizing radiation [37].

During advanced malignant transformation, the role of the AR becomes increasingly essential. ADT drives the development of active AR variants without ligand binding. After an initial successful ADT treatment phase, PCa becomes “androgen-independent”, but remains dependent on active AR signaling, which is conferred by various mechanisms that include AR point mutations, gene amplifications, activating AR splice variants, truncated AR variants, and upregulation of AR co-activators [38]. Point mutations accumulated at the dimerization interface, which probably reflect higher stability of the resulting monomers [19], and AR splice variants activate distinct transcriptional programs [39]. Very late-stage PCa is characterized by complete loss of the AR. This fatal development has been strongly advanced using highly effective AR inhibitors and degraders. Hence, treatment options that circumvent the development of this situation are urgently needed.

### 3.3. Crosstalk between AR and Other NRs/Transcription Factors

There are potential non-canonical AR interactions that have implications for PCa. Recent crystal structure data indicate a high flexibility of the core AR dimers which might form a head-to-tail or a head-to-head arrangement [40]. This non-canonical dimeric conformation theoretically opens many more spatial possibilities for potential interactions with other NRs or the cofactors that associate to form the transcriptionally active AR complex [19]. Atypical dimers are considered very likely to exist [27], though they are less prevalent than the canonical ones. Moreover, when AR function is strongly suppressed during ADT, atypical dimers can become essential and might even overtake AR function. There are likely direct consequences regarding physical NR interactions/competitions at the level of DNA [27] and to the regulation of subsets of genes. Indirect modes of pathway interference might include perturbations to the expression of the other NRs and/or their ligands [27]. All these possibilities might substitute for reduced AR function during ADT or might drive PCa growth per se.

**Figure 3:**
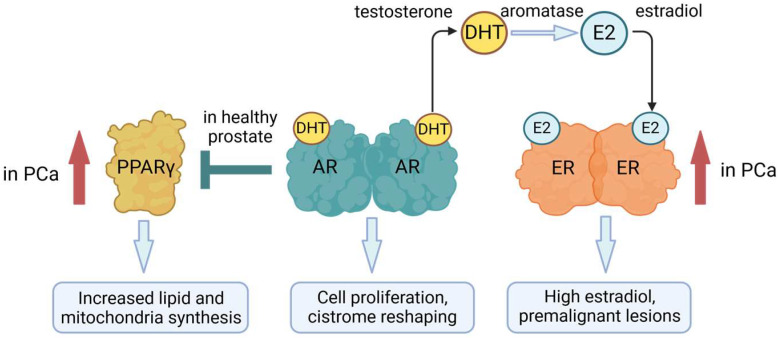
Androgen influences on prostate cancer (PCa). PCa is primarily an androgen-dependent disease. Estrogen receptors (ERs) play a role due to aromatase conversion of testosterone to estradiol. There is increased expression of peroxisome proliferator-activated receptor γ (PPARγ) during PCa, and AR inhibits the expression of PPARγ in healthy prostate. DHT, dihydrotestosterone. Created with BioRender.com.

There is a crosstalk between the AR and other NRs. RAR was upregulated post-androgen treatment of LNCaP cells; this was controlled by an ARE promoter element and led to upregulation of the epithelial growth factor receptor [41]. Upregulation of the glucocorticoid receptor was observed as an effect of ADT in PCa [42,43]. Though PCa is primarily an androgen-dependent disease, there is the interplay between the AR and ERs because androgens are aromatized to estrogens in adipose tissue, and the testosterone/estradiol ratio is essential to the development of PCa [44] (Figure 3). Low testosterone levels and high levels of estradiol lead to premalignant lesions providing evidence to support the importance of an imbalance to the ratio [45], and a high estradiol level in African-American men has been linked to a greater risk of developing PCa [46]. More recent work has focused on how estrogenic imprinting of the epigenome may contribute to PCa [47], and it has been argued that new compounds that target ERs might lead to therapeutic opportunities [48]. AR interacts with the PR regarding expression of the kallikrein-related peptidase 4 (KLK4). This protein is only expressed in PCa when there is expression of the AR and PRs. A hormone response element mediates gene expression whereby the progesterone receptor interacts directly with the gene promotor, and the interaction of AR is indirect [7].

Interactions between the AR and VDR have been reviewed elsewhere whereby AR-dependent and AR-independent actions of 1,25D3 play roles in inhibiting PCa cell line growth [49]. Importantly, for androgen-dependent LNCaP cells, AR stimulation via increased androgens suppressed the level of VDR, downregulation of AR increased VDR levels, and androgen withdrawal sensitized cells to the action of the vitamin D analogue 1α-hydroxyvitamin D5. The normal prostate cell line pRNS-1-1, which lost AR expression in culture, expressed VDR and was sensitive to 1α-hydroxyvitamin D5, and restoring AR expression led to resistance. Therefore, AR negatively regulates VDR levels [50]. AS3 (APRIN), a gene that is required for androgen-dependent growth arrest, has been reported to be a primary target for androgens and 1,25D3 [51]. Studies of LNCaP have also shown that 1,25D3 signaling negatively regulates AR signaling [52]. Whether the AR interacts directly with VDR is not known.

There is cooperativity between AR signaling and PPARγ, whereby AR inhibits the expression of PPARγ (Figure 3). At first, PPARγ was viewed as a tumor suppressor for PCa; agonists were shown to inhibit PCa cell growth, but this was then found to be PPARγ independent. Recently, it has been reported that there is increased expression of PPARγ during PCa progression with higher protein levels in advanced PCa compared to low-risk disease and benign hyperplasia [53]. Findings support the view that PPARγ supports PCa cell growth by increasing the machinery for lipid synthesis, including cutaneous fatty acid-binding protein (C-FABP) [54], fatty acid synthase (FASN), ATP citrate lyase (ACLY), and mitochondrial biosynthesis [55], bringing to attention that NRs regulate PCa metabolism. Upon loss of AR, antagonism of PPARγ may benefit the treatment of advanced stages of PCa. However, there is the need to take into consideration complexities relating to the different splice variants of PPARγ whereby increased PPARγ1 increased PCa tumorigenesis and FASN and ACLY expression whereas overexpression of PPARγ2 decreased LNCaP and PC3 proliferation and invasiveness [55]. 

Interaction of AR with tumor promoting signaling pathways has been observed, importantly with the Wnt pathway in CRPC [56]. Wnts are an ancient and conserved family of secreted glycoproteins that regulate the fate of developing cells [57]. Downstream of the binding of Wnts to frizzled receptors is the activation of the TF β-catenin [58], which plays a role in various cancers including PCa [59,60]. Recently, a direct interaction of AR with GATA3 has been shown to regulate a luminal epithelial phenotype in breast cancer [61].

## 4. Thyroid Hormone Receptor

### 4.1. The Functionalities of TRβ

Thyroid hormone (TH) is a primeval signaling pathway that arose in photosynthetic bacteria and algae. They take up iodate and convert it to iodide anions to create iodotyrosine [62]. This signaling molecule, which is present in all animal genera except arthropods, led to the evolution of TH synthesis [62,63]. THs are present in mollusks and worms onwards and regulate growth and development in non-bilaterian animals [63]. They provide a pro-survival stimulus to support metamorphosis in amphibians and lampreys and life-transitions in higher animals [64]. The effects of THs are dose, tissue, and developmental stage dependent [65].

THs exert their effect in vertebrates via TRs. The TR-coding cDNA was identified as a homologue to the avian erythroblastosis virus v-erb-A oncogene [66]. TR proteins are much shorter than the AR; TRα has 490 amino acids, and TRβ has 461 amino acids. However, a vast variety of isoforms is generated via alternative splicing. TRs are stimulated by thyroxine (T4) and its active derivative T3. They form homodimers or act as heterodimers in conjunction with RXRs [67]. There are two main TR isoforms: TRα and TRβ. TRα is predominantly expressed in the heart, bone, and brain, whereas TRβ is more abundant in the liver, kidney, and thyroid. TRα and TRβ seem to exert distinct tissue-dependent effects which are very likely complementary to each other, as revealed from studies of mutants [68]. Moreover, TRα and TRβ have opposing roles due to differences in their molecular properties and modes of action [69], based on diverse DNA binding affinities [70].

Unlike the AR, TRs belong to the class II NRs and reside constantly in the nucleus as a heterodimer with RXR. The canonical TR pathway involves binding of TR/RXR to thyroid response elements (TREs), which are, as mentioned above, direct repeats of two hexamer DNA-motifs spaced by four nucleotides (DR4) [24]. This binding mode is highly fine-tuned by the more specific DNA motifs [71]. Shuttling of T3 to the nucleus and binding to TRs attached to TRE elements in the genome leads to the loss of corepressors and the subsequent activation of gene transcription [72,73]. 

Further extensive analyses of TR DNA binding and potential ways of gene expression regulation have led to indications that there is gene regulation beyond canonical binding modes. TRs can bind to DNA as monomers [74], and dimeric variants have been described [19,75]. TRβ is the more interesting TR, and the gene that encodes it is located at chromosome 3. It seems to act in a more versatile manner, and several missense mutations are reported, which, as for the AR, map to the homodimer interface and might lead to altered ligand or coregulator binding. 

### 4.2. THs/TRβ and Prostate Cancer

As mentioned above, THs play a key role during development, and for vertebrates there are links to other endocrine systems as seen for zebrafish, birds, rodents, and dogs [76,77,78,79,80]. In mammals, close interactions between thyroid and gonadal hormone systems control sex-determination [81]. THs impinge on the maturation and functioning of the female [82] and male testicular development and function [79,83], including the stimulation of androgen release in the testis [84]. Thyroid disorders are associated with gonadal dysfunction, hypogonadism, and reduced prostate weight [85,86,87].

THs have long been suspected to be involved in the development of malignancies [88], whereby the maintenance of hypothyroxinemia was suggested to improve the survival of cancer patients [89]. Whether THs, including T3 and T4, play a role in supporting PCa growth, progression, and metastasis has remained largely unexplored. Albeit, THs influence the development and physiology of the human prostate [90] and the incidence of PCa, as seen from studies using a diet-induced regimen [91]. Epidemiological studies have linked low plasma T3 levels with a low incidence of PCa [92,93], and high T4 levels are significantly associated with an increased risk of any solid cancer, particularly PCa, lung, and breast cancer [92,94]. The latter finding led to the hypothesis that high TH levels correlate with tumor progression [95,96,97,98]. Several case reports have reported a correlation between low TH levels and slower cancer growth [72,99,100,101]. Studies of the availability of T3 for binding to TRβ support a role for T3 and TRβ in PCa. μ-Crystallin (CRYM) sequesters T3 in the cytosol to prevent T3 binding to nuclear TRβ [102], thereby controlling downstream target activation [96]. Our study showed that CRYM and TRβ show reciprocal expression in PCa tissue, whereby low CRYM represents a feature of metastatic PCa [103]. We and others have demonstrated that CRYM expression is deficient in hormone-refractory PCa patients [104,105] indicating reciprocal roles for CRYM and TRβ [106].

The literature is more extensive regarding the specific role of TRβ, perhaps reflecting a more significant role in cancer. TRβ is mainly described as a tumor suppressor [28] because expression is often reduced in human tumors due to deletions and epigenetic modifications [107]. A reduction has been linked to a poor prognosis in hepatocellular, renal, thyroid, and breast cancers [108,109,110]. TRβ expression is lost upon chromosomal 3p deletions, especially in breast and liver cancers [72]. In addition to somatic loss-of-function mutations that foster thyroid cancers, there is a high incidence of de novo inactivating TRα and TRβ mutations in hepatocellular, renal, and thyroid cancers [107] which may relate to selection pressures during tumor progression [111]. Loss of TRβ dysregulates several growth control pathways, leading to the exaggerated growth of various cancers [112]. 

In contrast to a tumor suppressor role for TRβ, TRα/TRβ1 double knockout mice develop fewer skin tumors [113], and enhanced TRβ expression has been detected in colon and head and neck cancers [114,115]. A recent study showed that low cytosolic and enhanced nuclear TRβ levels are indicators of a poor outcome in breast cancer [116], highlighting the importance of distinguishing between cytosolic and nuclear TRβ abundance, as recognized previously from studies of HeLa liver cancer cells [117]. Intriguingly, RXRs seem to have an anti-oncogenic role in PCa [118], providing tentative support to a tumor-promoting action of activated TRβ.

The literature regarding the influence of THs on cancer often presents a view of the clinical implications/morbidity relating to hypothyroidism [92,119,120]. THs stimulate the growth of many cancers, including breast, ovarian, pancreatic, hepatocellular, and renal cancer cells [121,122,123,124]. From in vitro studies, T3 supported the growth of lung, breast, ovarian, and squamous-cell cancer cells [72,96,125], and THs have a direct stimulatory effect on some key oncogenic signaling pathways, including the phosphatidylinositol-3-kinase (PI3K)- and extracellular signal-regulated kinase (ERK1/2)-mediated pathways.

The influences of THs on PCa are complex with outcomes from in vitro studies varying according to the cell lines tested and the TH and dose used. A non-physiological dose of T3 and T4 (7.7 μM) stimulated proliferation of the androgen-sensitive LNCaP cells, and an even higher dose (31 μM) was inhibitory. In this study, T3 and T4 decreased PC3 proliferation; T3 had no effect on DU145 cells; and T4 decreased proliferation. 3,3′-di-iodo-L-thyronine (T2) increased LNCaP proliferation and decreased that of PC3 cells [126]. Other works have reported that nM T3 stimulated the growth of LNCaP cells together with the expression of prostate specific antigen [127]. Long-term incubation led to an increased androgen binding capacity in the nucleus of LNCaP cells, suggesting that T3 might induce AR expression [128,129]. It is known that T3 and androgens act cooperatively to affect prostate-specific antigen (PSA) expression [103,127]. However, whether there is a direct interaction between AR and TRβ is unknown. Finally, T3, at 0.1 nM, has been reported to reduce LNCaP cell proliferation by driving cell senescence, and the known major mediators (p16^INK4A^ and p21^CIP1^) were not seen to be involved. In this study, T2 and T4 reduced growth to a lesser extent [130].

T3/TRβ-driven gene regulation has been analyzed in hepatocellular cancer to identify the proteins involved in tumor progression. T3-mediated upregulation of expression of the protease furin was seen for hepatoma cell lines, and the investigators concluded that this might enhance tumor metastasis [123,131,132,133]. TRβ has been shown to regulate cell metabolism and tumor-relevant genes. They include the hypoxia-inducible factor 1 subunit α [134], the CD44 stemness factor in the brain [135], cathepsin H (*CTSH* gene) which is a tumor invasive factor in HepG2 [133], and extracellular matrix proteins [123]. TRβ has been shown to effect mitochondrial respiration directly [136], which probably contributes to senescence, DNA damage, and oxidative stress.

Regarding in vivo studies, upregulation of mRNAs for TRβ together with other NRs and ONRs was observed for a PCa cell xenograft model [137]. Administering 6-n-propyl-2-thiouracil (PTU), to inhibit new TH production, reduced the growth of DU145 and PC-3 xenografts in mice [125] and reduced their growth in hypothyroid mice although enhanced aggressive behavior of hepatocellular and breast cancer cell xenografts was observed [107]. A high dose of T3 (2.5 μg/day for 6 weeks) reduced the growth of LNCaP xenografts in nude mice; these investigators also reported that T4 (100 nM) led to cultured LNCaP cells producing neurite-like projections [138].

### 4.3. Crosstalk between TRβ and Other NRs/Transcription Factors

**Figure 4:**
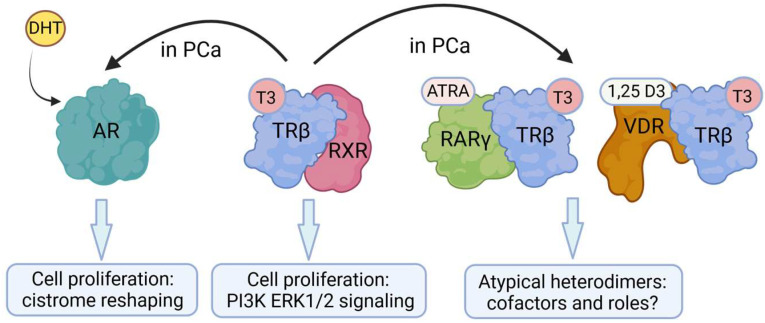
Crosstalk between thyroid hormone β (TRβ) and other nuclear receptors. 3,5,3′-triiodo-L-thyronine (T3) is known to increase dihydrotestosterone (DHT) production, and thyroid hormones increase the expression of androgen receptor (AR) and androgen synthesis enzymes. T3 and DHT strongly stimulate TRβ-mediated gene expression. TR/RAR heterodimers can regulate T3-mediated gene expression, and TR might interact directly with VDR. RAR, retinoic acid receptor; VDR, vitamin D receptor. ATRA, all-trans retinoic acid; 1,25D3, 1α,25-dihydroxyvitamin D3. Created with BioRender.com.

There is evidence to support the view that there is cross-regulation between THs and the androgen axis and that this occurs in cancer [139] (Figure 4). Exposure of the testes of the Western clawed frog (*Silurana tropicalis*) to T3 led to increased DHT production, and a low level of androgens in the serum of PCa patients correlated with decreased T3/T4 levels [140]. An androgen/TH interplay was recently observed in the PCa microenvironment to exert a cooperative tumor-promoting effect [141]. However, the above hormonal cross-regulation raises the question of whether the downstream effector NRs—in this case, TRβ and the AR—control the same or overlapping gene sets and processes [27]. TRβ/AR crosstalk was shown in silico via detailed promoter analysis of TH- and androgen-dependent genes, which revealed binding sites for both receptors (TRE and ARE sites) in the promoters of AR- and TR-regulated genes, indicating mutual or collaborative gene regulation [142]. Accordingly, TRβ-mediated gene expression is stimulated by both T3 and DHT robustly [143]. Other investigators have showed that THs increased the expression of AR and androgen synthesis enzymes [144]. These findings were corroborated by a recent transcription factor binding site study that detected an overlap of NRs binding to the same regulatory elements in PCa [145], opening the possibility of a mutual or collaborative TR/AR DNA-binding mode.

There is evidence to support the view that TRs interact with RARs. In 1993, Rosen and colleagues characterized a homologous 20-amino acid region conserved in TRβ, the RAR, and the VDR essential for heterodimerization [146]. They postulated the existence of various heterodimeric combinations to allow for enhanced variability and sensitivity of gene expression regulation. Other early studies also claimed that TRs and RARs form heterodimers, supported by overexpression and subsequent cell-free DNA-binding (EMSAs) and luciferase reporter assays in vitro [67,147,148,149]. Where RARs form heterodimers with TR, they have affinities for consensus and natural HREs like those for TR/RXR heterodimers. Therefore, TR/RAR heterodimers can regulate T3-mediated gene expression [149]. For amphibian limb blastemal and COS-transfected cells, ATRA was observed to mediate both RAR- and T3-mediated effects [150]. Stromelysin 3, a vital tissue remodeling protease, was found to be regulated by THs and ATRA due to the presence of both TREs and RAREs in the gene promoter [151]. For rat GH3 pituitary gland cells, ATRA has been shown to antagonize T3 action, presumably at a receptor level [152]. In addition to this complex hormonal crosstalk, T3 stimulation has been shown to regulate the expression of the ATRA synthesizing enzymes within mouse brain cells [153], underscoring the importance of hormonal crosstalk.

An important recent finding regarding PCa relates to the nuclear corepressor 2/silencing mediator (NCOR2) for RAR and TR. The expression of this corepressor is frequently altered in PCa and other cancers [154,155]. Reduced expression of NCOR2 has been shown to accelerate the failure of androgen deprivation. The investigators used the CWR22 xenograft model to show that a stable reduction in expression of NCOR2 accelerated the recurrence of disease post ADT [156].

In parallel to the early TR/RAR interaction studies, Schräder and colleagues showed that VDR might directly interact with TRβ, and that polarity directs gene expression [157,158]. Other workers identified interference by 1,25D3 and ATRA regarding TH regulation of glucocorticoid hormone expression, concluding that VDR–TR and VDR–RAR heterodimers act as competitors of TR–RXR and RAR–RXR complexes [159]. In contrast, others have failed to provide evidence for VDR–TR heterodimerization but showed that both VDR and TR compete for RXR binding [160], still highlighting a crosstalk between both receptors. Support to this view can be found in a study that showed that VDR heterodimerizes with RXR, but not with TRβ [161], and that a delicate balance of ligand availability and the limiting amounts of RXR, which would shift between TR and VDR, would affect the repression versus activation of various genes. Beyond the above findings, reports regarding crosstalk between TR and VDR have been limited. However, a very interesting crosstalk was described for adipocyte [162] whereby TRβ expression was controlled by 1,25D3 and vice versa, and that T3 regulating VDR gene expression in mouse photoreceptors has been reported [163].

There is a crosstalk between TRβ and other NRs. TRβ interacts with ER isoforms, resulting in flexible regulation of the consensus estrogen response element [164], and TRβ interaction with ERα has been shown to regulate mitochondrial activity [165]. TRβ interacts with PPARγ [166] and is then oncogenic [167]. Crosstalk between TRβ and the liver X receptor (LXR) has been identified for lipid metabolism-related genes and other physiological systems such as the central nervous system [168]. Regarding other TFs, TRβ interaction with the Wnt pathway is important based on direct interaction between TRβ and β-catenin [169]. Overall, TRβ interaction with the Wnt pathway for several organisms and tissues indicates the promotion of a stem cell phenotype [170]. Other crosstalk includes jun and fos oncogene activities, based on the abilities of TRs to inhibit AP-1 binding to DNA [171]. TRβ mediates repression of STAT5 activity [172], and overactivation of STAT5 signaling has been linked to an oncogenic TRβ variant in breast cancer [173].

Diverse findings bring to attention the complex role that TRβ plays in regulating the physiological status of cells. TRβ interacts with intracellular signaling pathways and with PI3K to play a role in the maturation of mouse hippocampal synapses [174]. There is TH regulation of steroid hormone-associated genes, for example, regulation of the expression of kidney androgen-regulated protein in the developing kidney [175] and T3 regulation of the expression of androgen receptor-associate protein 70 (ARA70) and sex hormone binding globulin (SHBG) in HepG2 cells [132,176]. TRβ modulates the function of the tumor suppressor p53, leading to differential regulation of p53-regulated genes [164]. Early studies revealed a connection between TR-mediated gene expression and changes to the expression of cell-cycle regulators [167,177]. Overexpression of TRβ in thyroid cancer cells led to activation of the RhoB signaling pathway and p21-induced cell-cycle arrest [178].

## 5. Retinoic Acid Receptors

### 5.1. The Functionalities of RARs

The three distinct RARs, namely RARα, RARβ, and RARγ, are encoded by different genes [179]. In addition, each RAR has isoforms, due to the presence of different promoters, alternative splicing, and use of non-AUG start codons [180,181,182,183]. RARγ is of particular interest regarding a potential new avenue for treating PCa (see later). The central portion of the RARγ protein, which contains the DNA and ligand binding domains, is encoded by seven axons. There are separate N-terminal exons for γ1 and γ2, and a major portion of the gene encodes several exons for untranslated regions of γ1 [184].

ATRA is the natural ligand of RARs and is used to control the transcription of target genes [185,186]. RARs form various heterodimers with RXRα, RXRβ, RXRγ, or homodimers, and the dimers bind to specific retinoic acid response elements (RAREs) within the promoters of genes. Without ATRA binding to RARs, the RAR is associated with RAR/RXR dimers to suppress gene expression due to RAR recruitment of transcription corepressors. ATRA binding to RAR leads to gene expression via the release of corepressors, the recruitment of coactivators [187], and the binding of further factors that include histone acetylase or histone methyltransferases, histone demethylases, and DNA-dependent ATPases [188,189].

Whether each RAR isoform has a distinct role is crucial to the consideration of their roles in cancer cells. Double knockout mice were needed to observe aberrant phenotypes that are associated with vitamin A deficiency indicating some functional redundancy [190,191,192]. Support to isoforms having distinct roles is that expression patterns are complex within embryonic and adult tissues. RARα is ubiquitous whereas RARβ and RARγ expression is either tissue-specific or regionalized [193,194]. Suggested roles for RARγ and RARβ are in the development of the central nervous system and the differentiation of adult stratified squamous epithelia, respectively [195]. RARγ expression is restricted to primitive cells during zebrafish embryo development [196], indicating a role in early morphogenesis as concluded from mouse studies [197]. 

RARα expression is ubiquitous within hematopoietic cells, with RARα2 increasing dramatically during myeloid cell differentiation [198], whereas RARγ is restricted to stem cells and primitive progenitors [199]. It is well known that activation of RARα promotes neutrophil differentiation of promyeloid cell lines [200,201] and normal progenitors [202]. These findings led to differentiation therapy for acute promyelocytic leukemia, with ATRA together with arsenic trioxide providing a cure [203]. Even so, RARα merely modules myelopoiesis [204], presumably by influencing progenitor cell decision-making to favor differentiation. In contrast, RARγ is needed to maintain hematopoietic stem cells because numbers were reduced in knockout mice [199]. Similarly, stem cell development was blocked when zebrafish embryos were treated with an RARγ agonist in the absence of exogenous ATRA [205]. 

Findings from molecular studies support the view that RARγ imposes stem cell stemness and/or restricts the temporal and/or spatial onset of the propensity of stem cells to differentiate. For embryonic stem cells, RAR/RXR dimers bound to gene loci that bind pluripotency TFs (SOX2, NANOG, and POU5f1) [187], and RARγ was required for chromatin epigenetic marks for transcription activation [206] and *Hoxa* and *Hoxb* gene reorganization [207]. The annelid worm (*Platyneris dumarilli*) RAR binds ATRA using a different pocket with low affinity for transactivation. It is, therefore, likely to be a permissive sensor and a suggested role is triggering of the spatially restricted onset of neurogenesis. High-affinity RARs have evolved at the base of chordates to sense complex ATRA gradients, and perhaps to regulate the *Hox* gene cluster [208].

### 5.2. RARs and Prostate Cancer

ATRA differentiation therapy for acute promyelocytic leukemia has not extended to PCa nor other cancers [209]. Albeit, and from immunohistology studies, increased expression of RARα and RARγ has been reported within high-grade PCa [210]. The LNCaP, PC-3, and DU145 prostate cancer cell lines express RARα and RARγ with LNCaP cells additionally expressing RARβ [211]. However, these cell lines are relatively insensitive to ATRA with the induction of growth arrest and apoptosis requiring a pharmacological amount (2 μM) [211,212]. 

Whether RARβ plays a role in PCa is uncertain. An association between the methylation status of RARβ2 and PCa risk has been reported [213], and a meta-analysis concluded that *RARβ* promotor methylation may be a correlate of PCa carcinogenesis. Further studies are needed to demonstrate a prognostic value because different methylation rates have been reported for PCa tissue [214]. That RARβ may play a role in controlling the proliferation of PCa has been argued from the finding that stable transfection of RARβ into the RARβ-negative PC-3 cells led to increased sensitivity to the combined use of an RARβγ-selective agonist (SR11262) and a potent vitamin D3 analogue regarding the inhibition of clonal growth. Increased sensitivity was proportional to the level of RARβ expressed [215].

RARγ expression is of prime importance to regulating the growth of PCa cells for the following reasons. PCa cells seem to have evolved to survive in a low-ATRA environment. ATRA levels for patients’ cells were close to the limit of detection (~1 ng/g tissue), whereas levels were up to eight-times higher in surrounding normal tissue and benign prostate hyperplasia [216]. This is important because nM concentrations transactivated RARγ, and 100-fold more was needed to activate RARα [201,217]. The proliferation of the PCa cell lines LNCaP, DU 145, and PC-3 was stimulated by a level of ATRA that was sufficient to activate RARγ but not RARα (10^−11^–10^−9^ M), and treatment with 10^−10^ M ATRA increased colony formation and the percentage of stem cell-like colonies. The RARγ agonist AGN205327 exerted the same effect as low doses of ATRA [218]. Therefore, the proliferation of PCa cells is highly dependent on the activity of RARγ. Findings from knockdown and knockout studies have provided further support to the view that RARγ promotes cancer cell proliferation, for example, from studies of colorectal and pancreatic cancer cells [219,220]. 

The activity of RARγ is also important to the survival of PCa cells. Treatment of flask cultures of patients’ cells and the LNCaP, PC-3, and DU145 cell lines with the selective RARγ antagonist AGN205728 was highly effective in driving growth arrest in the G1 stage of the cell cycle followed by necroptosis. A concentration of 5 nM AGN205728, close to its ED_50_ for RARγ of 3 nM, prevented colony formation by the cancer stem cell (CSC)-like cells of the PCa cell lines. AGN205728, therefore, targeted both CSC and non-CSC [218]. Normal prostate epithelial cells and non-neoplastic RWPE-1 cells were significantly less sensitive to the action of AGN205728 than patients’ PCa cells. The use of AGN194310 to antagonize all RARs was as effective as the RARγ-selective antagonist against patients’ cells and the PCa cell lines, and normal prostate epithelium cells were less sensitive [211,221,222]. Taken together, the findings support the development of an antagonist of RARγ for treatment of PCa.

RARγ is also an oncogene for various carcinomas [223]. Overexpression related to increased cell proliferation, rapid disease progression, and a poor prognosis has been reported for human colorectal cancer, cholangiocarcinoma, hepatocellular cancer, ovarian cancer, pancreatic ductal adenocarcinoma, renal cell cancer, and high-grade PCa. In keeping with the above-mentioned points, RARγ is an oncogene whose expression is downregulated by miR30a-5p [224], and this tumor suppressor miR is commonly at a low level in cancer cells. Acacetin targeting of the non-genomic actions of RARγ was effective against human hepatocellular cancer cell lines [225], and the specific aldehyde dehydrogenase inhibitors 673A, DIMATE, DEAB, NCT-501, silybin, and solomargine, which interfere with the endogenous synthesis of ATRA, were effective against lung, ovarian, prostate, and uterine cancer cells [223].

As considered above, RARγ regulates the behavior of multipotent stem/progenitor cells. RARγ is an oncogene, and the findings that overexpression within cancer cells correlated with a rapid disease progression and poor prognosis fits well with RARγ, ensuring the stemness of cancer stem cells. These cells are primarily responsible for both aggressive progression and disease relapse, and their frequency within a tumor varies from very minimal to up to 27% for melanoma [226]. This significant difference reflects the different types of cancer studied. Still, it may also reflect how well the method used to evaluate cancer stem cells within a tumor had efficiently measured their frequency as argued from studies of myeloma [226]. It is noteworthy that antagonizing RARγ killed PCa stem cells and all their offspring within flask-cultured cells, whereby offspring seem to have retained expression of RARγ. Antagonizing RARγ did not kill hematopoietic stem cells [201]. Like RARα, the action of RARγ is modulatory rather than obligatory to developmental processes. RARγ2 null mice were normal, and the fetuses of mice null for all RARγ isotypes were visibly normal. The latter mice exhibited growth deficiency, early lethality, and, interestingly, prostate squamous metaplasia [227]. Moreover, no adverse effects were seen when mice and rats were given substantial doses of a pan-RAR antagonist (BMS-18945) other than the inhibition of spermatogenesis, which was reversible [228,229]. The use of RAR antagonists seems to be safe in an adult organism. Why the cancer stem cells died via necroptosis when treated with very low doses of the RARγ and pan-RA antagonists is surprising and yet to be resolved.

### 5.3. Crosstalk between RARs and Other Transcription Factors

In addition to RARs competing with other nuclear receptor superfamily members for binding to RXRs and dimerization with TR (see above), they crosstalk with the AR. RARγ impacts androgen signaling because its expression level influences AR activity. ChIP-seq experiments in PCa cell lines showed that RARγ was bound to active chromatin and significantly overlapped with AR binding. Knockdown of RARγ in LNCaP cells affected the expression of genes associated with the AR response. Further experiments, using the HPr1-AR human prostate epithelial cell line, showed that RARγ knockdown resulted in a substantial reduction in the transcriptional response to DHT treatment with known AR target genes affected [230]. RARs crosstalk occurs in other ways as seen for ATRA and membrane receptor-provoked events. In 1990, investigators reported a negative cross-modulation between RARs and the activator protein 1, which regulates gene expression in response to cytokines [231].

β-catenin is particularly interesting regarding the crosstalk of RARγ with other TFs. RARγ physically interacts with β-catenin, as shown by co-immunoprecipitation studies. Overexpression in chondrocytes strongly inhibited β-catenin signaling, whereas silencing (RNA-mediated) of endogenous RARγ strongly increased signaling. The investigators proposed that unliganded RARγ would associate with β-catenin to inhibit its signaling [232]. Decreases in β-catenin target gene expression may be relevant to the ability of the RARγ antagonist to kill cancer cells. The interaction between RARγ and β-catenin is more complex because Wnt/β-catenin signaling regulates the expression of Yes-associated protein (YAP) as shown for colon cancer cells [233]. YAP is a coactivator of RARγ and acted via RAREs to reinforce stem cell traits within HT-29 colon cancer cells, including self-renewal. Conversely, the use of the pan-RAR antagonist BMS493 to silence RAR signaling downregulated the stem cell traits of HT29 and 5F31 cells, including their renewal capacity [234]. As seen for RARγ, YAP is an oncogene for several cancers, showing elevated expression in bladder, cervical, colon, gastric, non-small-cell lung, esophageal, and ovarian cancers [235]. In keeping with agonism of RARγ blocking stem cell development, YAP expression in human neural in vitro systems negatively correlated with neuronal differentiation and was seen to promote a neural rest/multipotent phenotype [236]. 

The role of the VDR, like that of RARα, is to promote cell differentiation, and there is crosstalk between the actions of RARα and the VDR. Studies of acute myeloid leukemia cell lines revealed that RARα regulated the expression of a VDR transcriptional variant originating in exon 1a. Unligated RARα repressed expression of the VDR gene whereby a high basal level of expression of RARα and a lack of RARα agonism correlated with repressed expression of the *VDR* gene. Downregulation of the level of RARα expression led to increased *VDR* gene expression [237]. Whilst RARs and VDRs preferentially bind DNA as heterodimers with RXR, a very early, and a perhaps ignored finding, was that VDR–RAR and VDR–RXR heterodimers can act functionally on direct repeat, palindrome, and inverted palindrome response elements [238].

## 6. Vitamin D Receptor

### 6.1. The Functionalities of VDRs

The two basic forms of vitamin D are D2 (ergocalciferol) and the main form D3 (cholecalciferol). Vitamin D is not a vitamin because vitamin D3 is made in human skin from 5,7-dehydrocholesterol via a non-enzymatic transformation and upon skin exposed to UV-B radiation [239]. Both forms of vitamin D are not biologically active, requiring conversion to the active forms 1α,25-dihydroxyvitamin D2 and 1,25D3, which have a systemic action like classic steroid hormones.

The composition of the VDR genomic region is very complex and covers ~100 kb located on chromosome 12 [240]. There are 14 exons with multiple enhancers regulating the tissue-specific expression of VDR proteins, with translation spanning from exons 2 to 9 [241,242]. In some individuals, there is a T-to-C polymorphism, which eliminates the most 5′-located ATG codon in exon 2, and translation is from the second in-frame ATG codon. This means that there are two variants of VDR with lengths of 424 and 427 amino acids [243]. Transcription regulation is complex for the VDR gene region that contains the six exons 1a to 1f because various tissues make alternative use of the exons and their corresponding promoters [241]. Three promoter regions have been identified for exons 1a–1f; exons 1a and 1d are regulated by the promoter upstream to exon 1a, and exons 1f and 1c have their upstream promoters. How the remaining exons are regulated remains to be elucidated [241,242,243]. 

There is widespread expression of VDRs within tissues. Most of the tissues responsive to 1,25D3 express transcripts originating from exon 1a and 1d. Transcripts that start from exon 1d give rise to a longer VDR protein, named VDR B1 [241,244]. The VDR plays a vital role in the endocrine control of calcium–phosphate homeostasis [245]. A transcript that originates in exon 1f is selectively expressed by tissues that play a role in calcium–phosphate homeostasis [241]. 

Here, we focus on the capacity of 1,25D3 to drive growth arrest, differentiation, and apoptosis of cancer cells. In this regard, the VDR binds to thousands of genomic loci to modulate the expression of hundreds of target genes. The VDR, after binding of 1,25D3, forms heterodimers with RXRα, RXRβ, and RXRγ to drive gene expression by modifying chromatin structure. The heterodimers bind to vitamin D response elements (VDREs) within the promotors of target genes (VDRE). The VDR also forms homodimers that interact with VDREs. Like RARs, the VDR is silent in the absence of a ligand due to the recruitment of corepressors. When a ligand is bound, gene expression occurs due to the recruitment of coactivators and eventually RNA polymerase II [246,247]. Secondary/indirect 1,25D target genes are regulated by transcription factors that are encoded by the primary target genes.

### 6.2. VDRs and Prostate Cancer

For over four decades, various studies have considered using 1,25D3 to treat multiple cancers [248]. Laboratory studies showed that 1,25D3 drives the growth arrest and apoptosis of a range of cancer cells, particularly PCa, breast cancer, and leukemia cells. However, the use of D vitamins to treat patients, in general, has been greatly hindered by their hypercalcemic action because increased blood calcium can lead to soft tissue calcification and increased bone resorption and can even be fatal [249]. Substantial concentrations of 1,25D3 are often used in in vitro and preclinical studies to achieve growth arrest and apoptosis of cancer cells. A recent appraisal of the literature has brought to attention that the positive outcomes from vitamin D clinical studies do not match up with those from in vitro and preclinical studies, whereby reaching an effective dosage in the clinic is likely to be an issue [250].

From mouse model studies of PCa, the growth of cells and metastasis were faster in mice that were fed a vitamin D3-deficient diet as compared to mice that were given 10,000 IU vitamin D3. Deprivation-mediated acceleration of growth and metastasis was attributed to epithelial-to-mesenchymal transition [251]. A concern regarding administrating vitamin D3 to increase 1,25D3 levels, or 1,25D3, is that 1,25D3 induces the expression of the 24-hydroxylase CYP24A1 which in turn readily catabolizes 1,25D3. Hence, in a PC-3 prostate xenograft model, mice were given 1,25D3 and a CYP24A1 inhibitor which resulted in enhanced inhibition of tumor cell growth [252]. 

A consensus view is that 1,25D3 can drive growth arrest and differentiation followed by apoptosis of many different cancer cell lines. Studies of prostate cancer cell lines show that various anticancer actions have been attributed to 1,25D3. For LNCaP cells, 1,25D3 enhanced apoptosis [253], up- and downregulated the expression of many genes [254], and inhibited AR signaling [52]. Reported 1,25D3-mediated effects on LNCaP, PC-3, and DU145 cells have included decreased invasiveness by the selective modulation of proteases [255] and disruption of glucose metabolism and the tricarboxylic acid cycle [256]. For LNCaP, the non-malignant prostate epithelium cells RWPE-1, and RMPE-2 cells (from RWPE-1 by ki-Ras transformation), an effect of 1,25D3 was to upregulate tumor suppressor miRNAs [257]. Additional 1,25D3 effects on RWPE-1 cells included an antioxidative action and the regulation of pathways such as Wnt, Notch, and NFκB1 [258,259]. 1,25D inhibited Wnt activity for patient-derived benign prostate epithelial organoids [260]. 

There has been a substantial effort to synthesize analogues of 1,25D3 that are more potent than the parent hormone and with a much-reduced calcemic action [261,262]. In particular, the PRI-1906 analogue has antiproliferative activity against PC-3 PCa cells [263]. As mentioned above, there has been a focus of attention on breast cancer, and the analogues PRI-1906, PRI-5201, and PRI-5202 were antiproliferative against human breast cancer cell lines [264,265]. PRI-5105, PRI-5106, and PRI-5202 were effective against human colon cancer cell lines [264,266].

1,25D3 does have effects on PCa, breast, and colon cancer cells, but of fundamental importance is whether any benefit might be seen from its use to treat patients. A prime consideration regarding PCa is that the aggressive cell lines are somewhat insensitive to the action of 1,25D3. It has been suggested that this unresponsiveness relates to an elevated nuclear expression of corepressors. In this case, 1,25D3 responsiveness is suppressed by a mechanism involving histone deacetylation, which might be overcome by using histone deacetylase inhibitors [267]. Even so, to date, 1,25D3 and their analogues have not provided an effective adjunct to chemotherapy for prostate cancer regarding a reduction in mortality in clinical trials. Though well-guided trials of vitamin D compounds are limited, there is limited data to support their use to treat or prevent PCa [268]. Whether newer 1,25D3 analogues that are much more potent than the parent hormone and with a negligible calcemic action can be developed and then used to provide some benefit as an adjunct to chemotherapeutics for PCa and other cancers remains to be seen.

Regarding an interest in the use of vitamin D to prevent PCa, an association has been reported from a meta-analysis between circulating 25-hydroxyvitamin D levels (a measure of vitamin D status) and a reduced risk of PCa [269]. However, this association remains unproven because of the variability of findings from both retrospective and prospective studies [270]. Different outcomes may be related to different levels of vitamin D supplementation. Even so, 12,927 persons received daily 2000 IU vitamin D3, and 12,944 persons received a placebo in the large VITAL trial, and there was not a significant reduction in the incidence of all cancers nor of PCa [271,272].

For all the NRs considered above, it is important to bear in mind that that the functioning of the immune system is under hormonal and NR control. To illustrate their importance, VDRs are expressed in most immune cells (antigen presenting cells and B and T cells), they make 1,25D3, and the VDR regulate many genes that are involved in immune functions [273]. Accordingly, 1,25D3 modulates innate and adaptive responses in many ways. They include support to the survival, proliferation, and differentiation of monocyte precursors/monocytes [274], regulation of the expression of complement factors [275], inhibition of the maturation of dendritic cells [276], and B cells [277] and natural killer and regulatory T cells are targets of the actions of 1,25D3 [278]. In principle, tumor initiation, growth, and metastatic progression can be controlled via cytotoxic innate and adaptive immunity [279]. Therefore, targeting NRs to enhance or decrease their activity is highly likely to have on effect on the immune function of cancer patients.

### 6.3. Crosstalk between VDRs and Other NRs/Transcription Factors

As considered above, the VDR largely form a heterodimer with RXR to regulate gene expression that controls cell differentiation and proliferation. There is crosstalk between the VDR and ER signaling as seen from studies of breast cancer cells. Estradiol upregulated the expression of the VDR in MCF-7 cells [280], and 1,25D3 induced expression of ERα in patients’ breast cancer cells to restore antiestrogen responsiveness. Induced expression was abrogated with a VDR antagonist [281]. There is crosstalk between the VDR and PPARγ. Binding of PPARγ to the VDR has been reported for human T47D breast cancer cells and PPARγ-mediated inhibition of VDR-mediated transactivation for T47D and LNCaP cells [282]. 1,25D3, via the VDR, inhibited both PPARγ expression and adipogenesis [283], and PPARγ is a primary target because there is a potent VDRE in the gene promoter [284]. However, crosstalk may be due to competition for binding to RXR [283]. VDR and PPARγ signaling play roles to regulate inflammation which is of importance to the tumor microenvironment. 1,25D3 promoted a switch from pro-inflammatory macrophages (M1) to anti-inflammatory (M2), and this effect was abolished by inhibiting the expression of the VDR and PPARγ [285]. From studies of melanoma cells, 1,25D3 treatment increased the expression of PPARα and PPARδ, and treatment with PPARα and PPARδ ligands increased VDR expression [286].

## 7. Drug Combination Therapies Involving NRs

The use of combination treatments in metastatic castration-resistant prostate cancer (mCRPC) to enhance outcomes has been reviewed elsewhere with the authors concluding that adoption is dismal despite supporting evidence [287]. There has been a focus on targeting AR signaling via two routes and targeting AR and other cellular processes. Phase III trials led to FDA approval of drugs to add to ADT as reviewed elsewhere [288]. The androgen synthesis inhibitor abiraterone and the AR antagonists enzalutamide, apalutamide, and darolutamide are currently used. Abiraterone (given with prednisone) and enzalutamide when administered alone as 1st-line treatments improved the survival of men with mCRPC, with PSA declining in up to 90% of patients [289,290]. A phase II trial optimized the use of abiraterone/prednisone together with enzalutamide to treat mCRPC, concluding that abiraterone followed by enzalutamide provided the greatest clinical benefit [291]. Abiraterone/prednisone together with enzalutamide is not indicated for men with rising PSA post enzalutamide treatment alone due to elevated liver enzymes and hypertension [292]. The poly(ADP-ribose) polymerase (PARP) plays a role in DNA repair and programmed cell death. Treatment of mCRP patients with the PARP inhibitors olaparib and abiraterone provided clinical benefit as compared with abiraterone alone [293], and from a systematic review, PARP inhibition is seen as an effective option [294]. 

Regarding dual NR targeting to treat PCa, the AR and GR share gene targets and induce PSA expression [295]. GR expression is high in DU145 and PC3 cells and low in AR^+^ LNCaP cells. GR may complement or substitute for AR functions, and when AR-mediated repression of GR was eliminated by ADT, GR expression was enhanced to confer antiandrogen resistance. A GR antagonist restored sensitivity to enzalutamide [42]. AR antagonism with specific GR antagonism could mitigate resistance to AR-targeted therapies. Unfortunately, the GR antagonists mefipristone, which binds to AR, and ORG34517 activate AR target gene expression. Efforts to find GR antagonists that lack this effect are promising, for example OP-3633 [296]. 

In breast cancer, combination therapies that target ER and other cellular processes have been studied extensively. For example, combining the ER antagonist fulvestrant with the CDK4/6 inhibitors palbociclib significantly improved outcomes in patients with ER-positive and human epidermal growth factor receptor 2 (HER2)-negative advanced breast cancer. Patients that received both drugs survived longer than those receiving fulvestrant and placebo, and the medium time to chemotherapy was extended [297]. How best to treat patients with aggressive triple-negative breast cancer (TNBC), whereby cells lack ER expression and make little HER2, remains a major challenge. It is interesting to note that treating two TNBC breast cancer cell lines with a VDR agonist and an AR agonist decreased cell viability; when combined, they appeared to be additive, and viability was decreased further when the agonists were combined with chemotherapeutic drugs. Paradoxically, the investigators also reported that the proliferation of most breast cancer cell lines was inhibited with AR antagonists [298]. There may be a benefit to dual targeting of AR and VDR in TNBC and other cancers.

PR activation influences ER signaling pathways, and PRs are differently expressed in breast cancer compared to normal tissue [299]. Dual targeting of PRs and ERs led to more effective inhibition of the growth of breast cancer cells. Mifepristone, when used at a low dose, and ORG-31710 are PR antagonists. They have an antiproliferative effect against breast cancer cell line cells, mainly regarding estradiol-stimulated growth, and ORG-3170 was more effective than mifepristone in regressing DMBA-induced tumors in rats. Studies using in vivo models have demonstrated additive effects when a combination of agents was used. Combining ORG-31710 with the antiestrogen tamoxifen was more effective in regressing tumors in rats than each agent alone. Clinical findings for the use of mifepristone, as 1st-, 2nd-, or 3rd-line treatment, showed promising results regarding breast cancer progression [300]. Even so, whether PR agonists and antagonists have efficacy in treating advanced breast cancer is yet unresolved with agents in trials or clinical use [301].

As mentioned above, there is the need to look towards evidence-based combination therapies for PCa, perhaps to add to ADT 1st-line treatment. Studies that have explored drug combination therapies for PCa and other cancers have provided support to focusing on NRs. Combinations can overcome resistance mechanisms and provide synergistic effects and should, therefore, be more effective than monotherapies. There are new promising avenues, for example the addition of an RARγ antagonist to kill CSCs and modulation of TH activity.

## 8. Concluding Remarks

The search for effective treatment options for advanced PCa, especially after the cessation of the effectiveness of hormonal therapy, has been ongoing for several decades. Management options have become scarce, the prognosis is poor, and further investigations are needed to overcome advanced disease. NRs are a promising class of signaling-effector targets regarding finding new treatments. Epidemiological and experimental findings affirm that NR signaling contributes to PCa pathogenesis and promotes progression. However, a deeper understanding of the molecular actions of NRs is required to define new diagnostic/prognostic and therapeutic targets. This includes the need to extend information regarding whether and how AR-, TRβ-, RARγ-, and VDR-mediated pathways converge in PCa to coregulate the expression of critical genes, for example, AR and the proteases kallikrein-related peptidase 3 and transmembrane-serine protease 2. On a mechanistic level, there is a need for a better understanding regarding whether ARs, TRβ, RARγ-, and VDRs undergo hitherto unknown interactions when having bound their ligands or relating to the recruitment of cofactors. The emergence of new and compelling omics technologies (ChIPseq, CUT&RUN, CUT&TAG, and qPLEX-RIME) enables transcription factor complexes to be determined and the regulated cistromes to be analyzed and compared. From rationales developed from such studies, using combinations of agents that target the actions of NRs may provide new therapeutic approaches to treating androgen-dependent and independent PCa, opening paths to treatments beyond androgen blockers. Drugs that interfere with hormones and RARγ and that activate VDR are well established and might be repurposed for PCa treatment.
